# Prevalence of possible idiopathic normal pressure hydrocephalus in older inpatients with schizophrenia: a replication study

**DOI:** 10.1186/s12888-020-02690-1

**Published:** 2020-06-01

**Authors:** Yuta Yoshino, Taku Yoshida, Hideo Morino, Masayuki Nakamura, Masao Abe, Hokuto Omachi, Saori Inoue, Yukiyo Miyoshi, Yumina Tachibana, Noriko Yamauchi, Naoya Takeda, Mutsuhiko Mizobuchi, Yuki Ozaki, Shinichiro Ochi, Junichi Iga, Shu-ichi Ueno

**Affiliations:** 1 Department of Neuropsychiatry, Molecules and Function, Ehime University Graduate School of Medicine, Shitsukawa, Toon, Ehime 791-0295 Japan; 2 Department of Psychiatry, Matsukaze Hospital, Doicho Irino 970, Shikokutyuo, Ehime Japan; 3 Department of Psychiatry, Juzen Yurinoki Hospital, Sumino Nittacho 1-1-28, Niihama, Ehime Japan; 4 Department of Neuropsychiatry, Shokokai Imabari Hospital, Takaichi 786-13, Imabari, Ehime Japan

**Keywords:** Schizophrenia, Normal pressure hydrocephalus, Cerebrospinal fluid tap test, Disproportionately enlarged subarachnoid space, Drug-induced extra-pyramidal symptoms scale

## Abstract

**Background:**

We recently reported that older patients with schizophrenia (SZ) show possible idiopathic normal pressure hydrocephalus (iNPH) more frequently than the general population. In this study, we estimated the prevalence of iNPH in a larger number of older SZ patients and explored useful examination values for diagnosis in the SZ population.

**Methods:**

We enrolled older inpatients with SZ (*n* = 39, mean age = 68.6 ± 7.7 years) from several psychiatric hospitals in Ehime, Japan and acquired brain imaging data using computed tomography. We evaluated three iNPH symptoms (dementia, gait disturbance, and urinary incontinence). In addition, we combined these data with our previous data to elucidate the relationship between iNPH and characteristics of SZ symptoms.

**Results:**

In total, five (12.8%) patients were diagnosed with possible iNPH. Evans’ index for patients with iNPH was significantly higher than for those without iNPH (*p* = 0.002). The number of disproportionately enlarged subarachnoid space hydrocephalus (DESH) findings was significantly higher in patients with iNPH than in those without iNPH (*p* <  0.001). Using combined data, Drug-Induced Extra-pyramidal Symptoms Scale (DIEPSS) subscales of gait and bradykinesia showed an increasing trend in the SZ with iNPH group.

**Conclusions:**

We reconfirmed that older inpatients with SZ experienced possible iNPH more frequently than the general population. We should pay attention to the DIEPSS subscales of gait and bradykinesia and DESH findings in addition to the three main symptoms of iNPH and Evans’ index so as to not miss SZ patients with iNPH.

## Background

The prevalence of physical comorbidities is higher in patients with schizophrenia (SZ) compared to the general population [1]. Common and serious comorbidities include metabolic syndrome [2] caused by second-generation antipsychotics [3] and/or cognitive impairment that leads to problems with adherence to those treatments [4]. To our knowledge, only limited studies have mentioned idiopathic normal pressure hydrocephalus (iNPH) among SZ patients, including one retrospective study [5], two case reports [6, 7], and one theoretical paper [8].

iNPH is treatable dementia with three main symptoms, which are dementia (psychomotor slowing and impaired attention, executive and visuospatial dysfunction), gait disturbance (shuffling, magnetic, and wide-based), and urinary incontinence [9, 10]. As for SZ symptoms, SZ patients also show cognitive impairments including psychomotor slowing, impaired attention, and executive function [11, 12]. Furthermore, antipsychotics often induce gait disturbance with Parkinsonism. Distinguishing iNPH symptoms from SZ symptoms or side effects of antipsychotics can be difficult, and it is possible to miss the iNPH symptoms in the daily clinical situations.

Surgical placement of a shunt is the only way to improve iNPH symptoms and prevent cerebrospinal fluid (CSF) storage. The CSF tap test is a valid method for diagnosis and for prediction of response to shunting [13–15]. Picascia et al. [16] suggested that shunt surgery in the early stage of iNPH may prevent progression of motor disturbances and cognitive impairment. In addition, adequate shunt surgery may improve the quality of life of iNPH patients [17].

The pathogenesis of iNPH is still unclear but there are considerable hypotheses based on previous evidence as Brautigam et al. reviewed [18]. There are four considerable etiologic and physiopathologic points (abnormal cerebrospinal fluid dynamics, vascular etiology, inflammation, and hereditary factors). As result of those, ventriculomegaly is consistent in iNPH patients. However, the ventriculomegaly can be seen in schizophrenia patients due to the increased volume of ventricles especially in elderly SZ patients [19]. Additionally, it is reported that the long-term medication of antipsychotics and benzodiazepine is associated with these brain structural changes [20]. The increase ventricles volume in SZ patients possibly mask the ventriculomegaly due to iNPH. In terms of risk factors for iNPH, diabetes mellitus (DM) is one of the possible risk factors, which can be involved for a vascular etiology [21, 22]. The risk of type 2 DM is 2- to 5-fold higher in SZ patients compared to general population [23]. In addition, it has been reported that metabolic syndrome itself (DM, hypertension, and hyperlipidemia) are also risk factors for iNPH [24–26]. We have hypothesized that these reasons increase the prevalence of iNPH in SZ population compared with the general population, and that is also higher the previously estimated prevalence in SZ population (3.1%).

In our preceding study, we reported the possibility of a higher prevalence (14.3%) of iNPH in Japanese SZ patients compared to the general population (0.51%) [27, 28]. However, the sample size of this study was relatively small (*n* = 21) and involved patients in a single hospital. In our current study, we recruited a larger number of older SZ inpatients from several hospitals and assessed both iNPH and SZ symptoms to reveal the prevalence of iNPH in SZ patients. Moreover, we combined these data with our preceding data to elucidate the relationship between iNPH and the characteristic SZ symptoms.

## Methods

### Subjects

We enrolled 39 SZ patients who were hospitalized in Matsukaze Hospital, Juzen Yurinoki Hospital, or Shokokai Imabari Hospital in Ehime, Japan from November 2017 to March 2018. SZ was diagnosed according to Diagnosis and Statistical Manual of Mental Disorder (DSM-5) criteria by at least two expert psychiatrists based on extensive clinical interviews and a review of medical records. The diagnosis of possible iNPH was conducted using the criteria proposed by the guidelines [29]. In detail, the required criteria are as follows: 1) Symptoms occur in the 60s or older, 2) More than one of the following clinical symptoms: gait disturbance, cognitive impairment, and urinary incontinence, 3) Ventricular dilation (Evans’ index > 0.3), 4) Clinical symptoms of iNPH cannot be completely explained by other neurological or non-neurological diseases, and 5) Preceding diseases possibly causing ventricular dilation are not obvious. The patients with a history of brain injury or stroke and those who used wheelchairs were excluded. All subjects were of unrelated Japanese origin and signed written informed consent forms approved by the institutional ethics committees of Matsukaze Hospital, Juzen Yurinoki Hospital, Shokokai Imabari Hospital, and Ehime University Graduate School of Medicine.

### Assessment of symptoms of SZ and iNPH

Symptoms of SZ were assessed by the Positive and Negative Symptom Scale (PANSS) (each item is scored on a 1–7 scale) [30]. For the combined data analysis, the Brief Psychiatric Rating Scale (BPRS) score was estimated by PANSS according to a previous paper [31]. We evaluated antipsychotic-induced extrapyramidal symptoms using the Drug-Induced Extrapyramidal Symptoms Scale (DIEPSS) [32].

We evaluated three major iNPH symptoms including gait disturbance, cognitive disturbance, and urinary disturbance with the same tests as in our preceding study [28]. The iNPH grading scale (iNPHGS) included three items: gait disturbance (GS-Gait), cognitive impairment (GS-Cognition), and urinary incontinence (GS-Urine). Patients and caregivers were interviewed to assess iNPHGS [33].

Timed Up and Go (TUG) [34] and the 10-m walking test [35] were used to assess gait disturbances. We conducted those tests twice, and the mean scores were calculated. Gait disturbances were also assessed by the Gait Status Scale (GSS) [33].

Cognitive impairment was evaluated with the Mini-Mental State Examination (MMSE) [36]. Caregivers (the attending nurse in this study) assessed neuropsychiatric symptoms with the neuropsychiatric inventory (NPI) [37].

Imaging data were obtained using a computed tomography (CT) scanner. We defined results for the Evans’ index > 0.3 as abnormal [21].

### Genotyping

Genotyping of single-nucleotide polymorphisms (rs429358 and rs7412) of apolipoprotein E (*APOE*) was conducted using the TaqMan 5′-exonuclease allelic discrimination assay (Assay ID: rs429538; C___3084793_20 and rs7412, respectively, Applied Biosystems) using the StepOnePlus real-time PCR system (Applied Biosystems). Genotyping call rates were 100.0% (rs429358) and 100.0% (rs7412). We found no deviation from the Hardy-Weinberg equilibrium in each examined single-nucleotide polymorphism in the patients (*p* > 0.05). The *APOE* isotype-related genotypes are combinations of the *APOE* ε2, ε3, and ε4 alleles derived from the two genotypes of rs429358 (T334C) and rs7412 (C472T): ε2, 334 T/472 T; ε3, 334 T/472C; and ε4, 334C/472C. The ε4 genotype is a risk factor for Alzheimer’s disease (AD) [38].

### Statistical analysis

Statistical analyses were conducted with EZR version 1.36 [39]. The Shapiro-Wilk test was used to test for normality. Comparisons between no iNPH and possible iNPH patients were conducted for age, body mass index (BMI), onset of age, duration of illness, chlorpromazine (CP) equivalent dose, PANSS, DIEPSS, iNPHGS, TUG, 10-m walking test, GSS, MMSE, NPI, and Evans’ index. Student’s *t*-test was used for normally distributed data, and the Mann-Whitney U test was used for those not normally distributed. The average DIEPSS subscales were analyzed with Student’s *t*-test or the Mann-Whitney U test with Bonferroni correction. The differences in sex, smoking status, hypertension, hyperlipidemia, diabetes mellitus, disproportionately enlarged subarachnoid space hydrocephalus (DESH), and *APOE* genotypes were analyzed using the Fisher’s exact test. Correlations for each parameter were analyzed by determining the Pearson’s correlation coefficient. Statistical significance was defined at the 95% level (*p* = 0.05).

## Results

### No iNPH vs. possible iNPH groups

**Fig. 1:**
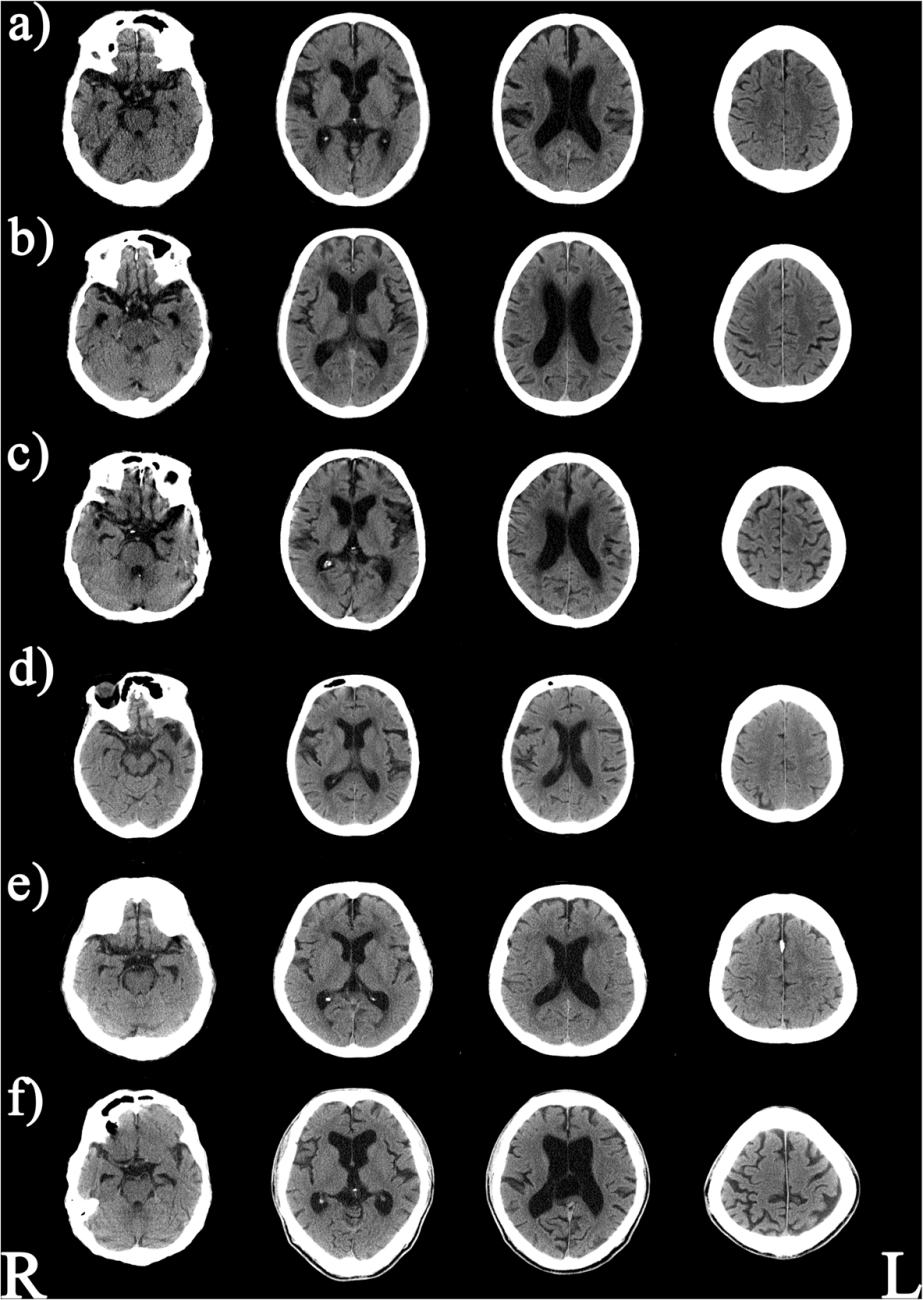
Shown are computed tomography images of **a** an 84-year-old male; **b** a 78-year-old male; **c** a 74-year-old male; **d** a 64-year-old female; and **e** a 61-year-old male with idiopathic normal pressure hydrocephalus. Panel **f** shows a computed tomography image of a 62-year-old male with secondary normal pressure hydrocephalus with a mass in the lateral ventricle. All subjects have features of an enlarged Sylvian fissure associated with ventriculomegaly and compression of high convexity. Several subjects (**a**, **b**, **d**, and **e**) have disproportionately enlarged subarachnoid space hydrocephalus findings

All the possible samples were recruited except samples meeting exclusion criteria or who refused this study. Finally, 38 subjects were collected, and those characteristics and results of their examinations are shown in Table 1. In total, five of 38 (12.8%) patients were diagnosed with possible iNPH. We excluded one patient with secondary NPH from statistical analysis. In this case, NPH was thought to be caused by a mass that was detected with CT (Fig. 1f). Evans’ index in patients with iNPH was significantly higher than in those without iNPH (*p* = 0.002). The number of DESH findings was significantly higher in patients with iNPH than in those without iNPH (*p* <  0.001).

**Table 1:** Demographic and clinical data of patients with possible iNPH and without iNPH (no iNPH)

Characteristics	No iNPH (*n* = 33)	Possible iNPH (*n* = 5)	*p* value
Sex, male	21	4	0.643
Age (years)	68.3 ± 7.4	72.2 ± 9.6	0.352
BMI (kg/m^2^)	21.5 ± 2.3 (29)	23.4 ± 2.7 (4)	0.133
Smoking	9	0	0.555
Hypertension	7	1	1.0
Hyperlipidemia	9	1	1.0
Diabetes Mellitus	1	0	1.0
Age of onset (years)	23.2 ± 8.1	24.8 ± 7.3	0.632
Duration of illness (months)	45.0 ± 7.9	47.4 ± 13.2	0.436
CP equivalent	820.9 ± 500.9	741.2 ± 591.4	0.863
Drug administration			
Anticholinergics	10	2	1.0
Benzodiazepines	23	2	0.342
Antidepressants	0	0	–
Mood stabilizers	13	0	0.342
PANSS–total	89.4 ± 15.4	87.0 ± 18.9	0.931
PANSS–positive	21.6 ± 5.1	18.4 ± 5.7	0.363
PANSS–negative	24.3 ± 6.9	24.8 ± 7.0	0.914
PANSS–general	43.5 ± 9.2	43.8 ± 6.7	0.762
DIEPSS	5.8 ± 5.0	10.4 ± 5.0	0.062
iNPHGS			
GS-Gait	0.91 ± 1.2	2.2 ± 1.8	0.082
GS-Cogn	1.9 ± 1.2	2.4 ± 1.5	0.471
GS-Urin	1.0 ± 1.6	1.6 ± 1.8	0.425
TUG	14.7 ± 6.6 (26)	10.6 ± 2.7 (3)	0.267
10-m walking test	14.0 ± 4.9 (26)	15.3 ± 7.3 (3)	0.858
GSS	3.2 ± 3.1 (29)	5.0 ± 2.6 (3)	0.192
MMSE	19.6 ± 6.3 (31)	15.6 ± 8.3	0.281
NPI	15.4 ± 11.4	8.0 ± 7.9	0.160
Evans’ Index	0.27 ± 0.04	0.34 ± 0.03	0.002
DESH	2	4	< 0.001
*APOE* ε4+/ε4−	8/25	1/4	1.0

Values denote number (%) or mean ± standard deviation

*BMI* body mass index, *CP equivalent* chlorpromazine equivalent, *DESH* disproportionately enlarged subarachnoid space hydrocephalus, *DIEPSS* Drug-Induced Extra-pyramidal Symptoms Scale, *iNPH* idiopathic normal pressure hydrocephalus, *iNPHGS* idiopathic Normal-Pressure Hydrocephalus Grading Scale, *GS-Gait* iNPHGS for gait, *GS-Cogn* iNPHGS for cognition, *GS-Urin* iNPHGS for urinary function, *TUG* Timed Up-and-Go test, *GSS* Gait Status Scale, *MMSE* Mini-Mental State Examination, *PANSS* Positive and Negative Syndrome Scale, *NPI* neuropsychiatric inventory

Gender (*p* = 0.643), age (*p* = 0.352), BMI (*p* = 0.133), smoking status (*p* = 0.555), hypertension (*p* = 1.0), hyperlipidemia (*p* = 1.0), diabetes mellitus (*p* = 1.0), age of onset (*p* = 0.632), duration of illness (*p* = 0.436), CP equivalent (*p* = 0.863), use of anticholinergics (*p* = 1.0), use of benzodiazepine (*p* = 0.342), use of mood stabilizers (*p* = 0.342), PANSS-total (*p* = 0.931), PANSS-positive (*p* = 0.363), PANSS-negative (*p* = 0.914), PANSS-general (*p* = 0.762), DIEPSS (*p* = 0.062), iNPHGS-Gait (*p* = 0.082), iNPHGS-Cogn (*p* = 0.471), iNPHGS-Urin (*p* = 0.425), TUG (*p* = 0.267), 10-m walking test (*p* = 0.858), GSS (*p* = 0.192), MMSE (*p* = 0.281), NPI (*p* = 0.160), and *APOE* genotype (*p* = 1.0) were not significantly different (Table 1) between groups. CT data of possible iNPH and secondary NPH patients are shown in Fig. 1.

### Analysis of combined data from this and the previous study

The demographics of the combined data and the results of statistical analysis are shown in Table 2. Eight of 59 (13.6%) SZ patients were diagnosed with possible iNPH as a comorbidity. The average DIEPSS (*p* = 0.006), iNPHGS-Gait (*p* = 0.015), GSS (*p* = 0.009), and Evans’ index (*p* <  0.001) were significantly higher in patients with possible iNPH compared to patients without iNPH. The proportion of patients with DESH was higher in the possible iNPH group than in the no iNPH group (4/51 vs. 5/8, *p* = 0.001).

**Table 2:** Demographic and clinical data of patients with possible iNPH and without iNPH (no iNPH) with combined data (present and preceding study)

Characteristics	No iNPH (*n* = 51)	Possible iNPH (*n* = 8)	*p* value
Sex, male	29	6	1.0
Age (years)	69.0 ± 6.8	74.0 ± 8.5	0.092
BMI (kg/m^2^)	20.5 ± 2.5 (46)	21.9 ± 2.9 (7)	0.294
Smoking	13	0	0.176
Age of onset (years)	27.1 ± 12.9	25.0 ± 7.9	0.912
Duration of illness (months)	41.6 ± 11.3	53.9 ± 21.3	0.069
CP equivalent	717.0 ± 493.5	655.4 ± 497.0	0.842
Drug administration			
Anticholinergics	20	2	0.698
BPRS	41.8 ± 13.2	43.3 ± 13.4	0.765
DIEPSS	5.9 ± 4.5	10.9 ± 4.2	0.006
iNPHGS			
GS-Gait	0.96 ± 1.1	2.3 ± 1.5	0.015
GS-Cogn	1.8 ± 1.2	2.5 ± 1.2	0.107
GS-Urin	0.96 ± 1.1	1.8 ± 1.7	0.178
TUG	16.4 ± 9.0 (44)	24.2 ± 14.9 (6)	0.376
10-m walking test	15.2 ± 6.1 (44)	25.2 ± 14.2 (6)	0.064
GSS	3.1 ± 3.2 (47)	7.7 ± 4.8 (6)	0.009
MMSE	18.9 ± 5.5 (48)	16.1 ± 7.2 (7)	0.317
NPI	15.8 ± 11.4	16.6 ± 22.3	0.451
Evans’ Index	0.28 ± 0.04	0.37 ± 0.07	< 0.001
DESH	4	5	0.001
*APOE* ε4+/ε4−	11 / 40	2 / 6	1.0

Values denote number (%) or mean ± standard deviation

*BMI* body mass index, *CP equivalent* chlorpromazine equivalent, *BPRS* Brief Psychiatric Rating Scale, *DIEPSS* Drug-Induced Extra-pyramidal Symptoms Scale, *iNPH* idiopathic normal pressure hydrocephalus, *iNPHGS* idiopathic Normal-Pressure Hydrocephalus Grading Scale, *GS-Gait* iNPHGS for gait, *GS-Cogn* iNPHGS for cognition, *GS-Urin* iNPHGS for urinary function, *TUG* Timed Up-and-Go test, *GSS* Gait Status Scale, *MMSE* Mini-Mental State Examination, *NPI* neuropsychiatric inventory

We found no significant differences in sex (*p* = 1.0), age (*p* = 0.092), BMI (*p* = 0.294), smoking (*p* = 0.176), age of onset (*p* = 0.912), duration of illness (*p* = 0.069), CP equivalent (*p* = 0.842), use of anticholinergics (*p* = 0.698), BPRS (*p* = 0.765), iNPHGS-Cogn (*p* = 0.107), iNPHGS-Urin (*p* = 0.178), TUG (*p* = 0.376), 10-m walking test (*p* = 0.064), MMSE (*p* = 0.317), NPI (*p* = 0.451), or *APOE* genotype (*p* = 1.0) between groups.

Subsequently, we analyzed the subscales of DIEPSS (Table 3). None of the subscales were significantly different between groups after Bonferroni correction (Gait: *p* = 0.021; Bradykinesia: *p* = 0.015; Sialorrhea: *p* = 0.531; Rigidity: *p* = 0.063; Tremor: *p* = 0.376; Akathisia: *p* = 0.021; Dystonia: *p* = 0.145; Dyskinesia: *p* = 0.468; Global: *p* = 0.011).

**Table 3:** Subscales of the Drug-Induced Extra-pyramidal Symptoms Scale (DIEPSS)

Items	No iNPH (*n* = 51)	Possible iNPH (*n* = 8)	*p* value
Gait	1.0 ± 1.4	2.0 ± 1.9	0.021
Bradykinesia	1.2 ± 1.1	1.8 ± 0.8	0.015
Sialorrhea	0.6 ± 0.9	0.4 ± 0.5	0.531
Rigidity	0.6 ± 0.9	1.6 ± 1.5	0.063
Tremor	0.7 ± 1.0	1.2 ± 1.6	0.376
Akathisia	0.1 ± 0.4	0.6 ± 0.5	0.021
Dystonia	0.1 ± 0.3	0.4 ± 0.5	0.145
Dyskinesia	0.2 ± 0.7	0.6 ± 0.9	0.468
Global	1.2 ± 0.9	1.8 ± 0.4	0.011

The average differences in the DIEPSS subscales were analyzed with the Student’s *t*-test or Mann-Whitney U test with Bonferroni correction (statistical significance: *p* < 0.006)

None of the MMSE subscales were significantly different between groups after Bonferroni correction (Orientation in time: *p* = 0.012; Orientation in place: *p* = 0.968; Registration: *p* = 0.048; Serial-7: *p* = 0.124; Repetition: *p* = 0.189; Three-stage command: *p* = 0.724; Reading: *p* = 0.179; Recall: *p* = 0.443; Naming: *p* = 0.023; Writing: *p* = 0.179; Construction: *p* = 0.423, Table 4).

**Table 4:** Subscales of the Mini-Mental State Examination (MMSE)

Items	No iNPH (*n* = 48/51)	Possible iNPH (*n* = 7/8)	*P* value
Orientation in time	3.3 ± 1.6	1.6 ± 1.3	0.012
Orientation in place	3.7 ± 1.3	3.7 ± 1.5	0.968
Registration	2.9 ± 0.4	2.3 ± 1.3	0.048
Serial-7	1.1 ± 1.4	1.7 ± 1.6	0.124
Repetition	0.9 ± 0.3	0.7 ± 0.5	0.189
Three-stage command	2.1 ± 1.0	2.3 ± 1.0	0.724
Reading	0.8 ± 0.4	0.9 ± 0.4	0.179
Recall	1.2 ± 1.1	0.9 ± 0.9	0.443
Naming	2.0 ± 0.2	1.7 ± 0.5	0.023
Writing	0.6 ± 0.5	0.3 ± 0.5	0.179
Construction (cube-copying)	0.3 ± 0.5	0.1 ± 0.4	0.423

The average differences in the MMSE subscales were analyzed with the Student’s *t*-test or Mann-Whitney U test with Bonferroni correction (statistical significance: *p* < 0.0045)

## Discussion

This study has three major findings.

First, we found that five of 38 (12.8%) subjects had possible iNPH, which is similar to our preceding study (14.3%) [28]. The total rate from the combined data was eight of 59 (13.6%), which is approximately 4–20 times higher than that of the Japanese general population (0.5–2.9%) [27, 40, 41]. Consistently, Vanhala et al. [5] estimated that SZ patients were present 3 times more frequently among Finnish iNPH patients compared to the general aged population. However, this prevalence was estimated from already diagnosed iNPH patients. SZ patients hesitate to visit not only psychiatrists but also doctors for physical problems for several reasons. One reason is that they do not recognize physical symptoms due to the lack of feeling unpleasant stimulation such as pain [42]. The second reason is that barriers to receiving health care exist such as a lack of access to health care, lack of integration of medical and mental health systems, and denial of illness [43]. Indeed, several patients with possible iNPH denied undergoing a CSF tap test after informing them of the diagnosis and how to treat the illness. In some cases, we could not determine a diagnosis of possible iNPH due to the patient’s cognitive dysfunction, inability to access the hospital for treatment, and their prognosis. In the future, we must find a way that allows SZ patients with possible iNPH to easily receive the required medical care. For these reasons, we think that the actual prevalence of iNPH in SZ patients is higher than estimated by Vanhala et al. [5].

Second, we found significant changes in CT findings of DESH in addition to Evans’ index between the group with possible iNPH and the group with no iNPH. An Evans’ index greater than 0.3 is one of the required diagnosis criteria [29], but an Evans’ index greater than 0.3 was found in some SZ patients without iNPH. The Evans’ index generally reflects ventriculomegaly [44]. However, enlargement of the lateral ventricles occurs with non-specific global brain atrophy associated with aging even in healthy subjects and those with neurodegeneration [45]. Patients with chronic SZ also have overall brain atrophy and enlargement of ventricular volumes [46]. We should pay more attention to the use of the Evans’ index for diagnosis of iNPH in SZ patients. DESH is a reliable marker of diagnosis [47, 48] and shunt responsiveness [49]. In addition, several reports also indicate the usefulness of the callosal angle for diagnostic [50, 51] and shunt responsiveness [52–54]. We think that using DESH findings in addition to the Evans’ index for making a diagnosis of iNPH in SZ patients is better than using the Evans’ index only.

Third, we explored useful examination values for diagnosis of iNPH by considering three main symptoms, which are dementia, gait disturbance, and urinary incontinence [9, 10]. In terms of dementia, we found no significant difference in MMSE or iNPHGS–Cogn between the iNPH and no iNPH groups. The progressive cognitive dysfunction in SZ may affect these results [55]. Moreover, the main purpose of MMSE is to understand general cognitive dysfunction. Especially in iNPH cognitive dysfunction, psychomotor slowing, executive dysfunction, and apathy are well-known symptoms [35, 56], and these symptoms are typically characterized as frontal lobe impairment [57]. These findings are consistent with single photon emission computed tomography and positron emission tomography studies [58, 59]. As the previous study showed [60], the use of frontal cognitive assessments such as word fluency, Trail Making Test A, and the Frontal Assessment Battery for understanding the cognitive dysfunction in SZ with iNPH patients is better than MMSE. In terms of side effect of drugs, antipsychotics could induce cognitive dysfunction as a side effect. Especially, clinicians should pay attention to SZ patients who take antipsychotics that have an anticholinergic effect [61, 62]. In addition, it has been reported that the tapering of benzodiazepine and anticholinergic rescue cognitive function [63, 64]. Regarding urinary incontinence, iNPHGS-Urin was not significantly changed in the iNPH group compared to the no iNPH group. The risk of urinary incontinence in SZ patients is 1.78-fold higher than that in non-SZ patients [65]. Even here, it has been reported that one of the reasons for urinary dysfunction including urinary inconsistent and retention is the anticholinergic effect in antipsychotics [66]. The fact that urinary incontinence occurred during SZ treatment may possibly mask urinary incontinence as an iNPH symptom. From the viewpoint of gait disturbance, we found significant changes in the total score of DIEPSS, iNPHGS–Gait, and GSS by analyzing combined data. Here, our question is how we can distinguish gait disturbance that is part of iNPH from that caused by side effects of antipsychotics. The most common mechanism of drug-induced parkinsonism is the blockade of D2 receptor in the striatum. To examine that point, we additionally analyzed the subscales of DIEPSS. We found that subscales of gait and bradykinesia tended to increase in the iNPH with SZ group even though the difference did not reach statistical significance after Bonferroni correction. We consider that SZ patients with a high score for gait and bradykinesia and a low score for other parameters in DIEPSS should be suspected as having iNPH. Furthermore, older patients generally have decreased liver and kidney function, and they easy to get the side effects of antipsychotics [67]. To review the antipsychotics prescription toward older SZ patients will help us to distinguish the gait disturbance of iNPH symptoms from that of side effect.

In addition, we would like to mention the suspected overlapping pathogenesis between SZ and iNPH even though this will be speculative. One autopsy study revealed a high overlapping rate (8/9, 89%) between iNPH and AD [68]. To examine this point, we did the genotyping for *APOE* and unable to find the difference of ε4+ frequency between iNPH with SZ and iNPH without SZ group. However, ε4+ frequency was higher regardless of iNPH in this study (non-iNPH vs iNPH = 32% vs 25%) than that of Japanese SZ population (11.1–18.3%) [69–71]. We think that it is hard to conclude this point due to the small sample size of this study. In terms of CSF marker, elevated IL-6 and -8 were reported in patients with iNPH [72, 73]. These findings are the same as SZ population as reviewed in [74]. These elevated cytokines are thought to reflect the inflammatory changes of iNPH pathogenesis in the brain. As mentioned in introduction section, vascular etiology is the consistent risk factor for developing iNPH. Among SZ patients, metabolic syndrome is a common comorbidity [2], which may be the one reason to indicate the high prevalence of iNPH in SZ population.

Our study has a few limitations. First, two of three possible iNPH patients declined to undergo the gait disturbance examinations (TUG, 10-m walking test, and GSS). This may have affected our observation of no significant difference in examinations between the no iNPH and possible iNPH patients in this study, even though our preceding study showed significant differences in the TUG and 10-m walking test^20^. Second, selection bias may have been present in this study because we recruited only inpatients. That is extremely limited SZ population, which is relatively far from general SZ population. Further studies with larger samples including outpatients are needed to reach a conclusion about the prevalence of iNPH and to reveal which examination is useful for diagnosis. Finally, we could not completely exclude other comorbid conditions, especially AD. Several studies have reported that patients with NPH had a high comorbid ratio of neuropathological change of AD by conducting biopsy at the time of shunt surgery (7/21 [33%], [75]; 23/55 [42%], [76]).

## Conclusions

The prevalence of iNPH may be higher in older SZ patients than in the general population. We should pay more attention to the three main symptoms of iNPH when we treat older SZ patients. The DIEPSS subscales of gait and bradykinesia and DESH findings may be useful for diagnosing possible iNPH in SZ patients. Psychiatrists are responsible for diagnosing and treating iNPH in older SZ patients without overlooking this possibility. In daily medical care of SZ patients, clinicians possibly could find the early stage of iNPH by paying attention to those symptoms and provide the early treatment for iNPH patients in the older SZ population. To verify the iNPH symptoms clearly in older SZ patients, we had better to decrease benzodiazepine and anticholinergics, and optimize the doses of antipsychotics.

## Data Availability

The datasets used and/or analyzed during the current study are available from the corresponding author on reasonable request.

## Abbreviations

AD: Alzheimer’s Disease
APOE: Apolipoprotein E
BPRS: Brief Psychiatric Rating Scale
CT: Computed Tomography
DIEPSS: Drug-Induced Extrapyramidal Symptoms Scale
DSM-5: Diagnosis and Statistical Manual of Mental Disorder
GSS: Gait Status Scale
iNPH: idiopathic Normal Pressure Hydrocephalus
iNPHGS: iNPH Grading Scale
MMSE: Mini-Mental State Examination
NPI: NeuroPsychiatric Inventory
PANSS: Positive And Negative Symptom Scale
SZ: Schizophrenia
TUG: Timed Up and Go
